# Items of General Interest

**Published:** 1915-12

**Authors:** 


### Items of General Interest.

In a paper read before the Southern Medical Association, Dr. L. M. Graves, of Galveston, advocated the emasculation of all idle negroes in the South. Dr. Graves declared vagrant negroes to be a serious menace to the health of the entire section, because many of them are insanitary, immoral, licentious and unmindful of the importance of health measures.

---

The *Wise County Bulletin* says: "With Dr. Bacon Saunders, President of the Southern Surgical and Gynecological Association; Dr. Joe Becton, President of the Medical Association of the Southwest, and Dr. Holman Taylor, Vice-President of the Southern Medical Association, and Dr. Wm. Rodman, President of the American Medical Association, only a few years past being a practitioner at Abilene, the Texas medical profession has much to be proud of."

Yes, the outside world is beginning to recognize that Texas grows big, capable men as well as cotton and longhorn cattle.

---

After convincing the physicians that she had swallowed a table knife in a delirium, a Chicago woman was operated on and the knife removed from her stomach. The knife was corroded and saw-edged from the action of the acids of the stomach.

---

Dr. Rupert Blue, Surgeon General of the United States Public Health Service, was in Dallas last week attending the Southern Medical Association. He said it had been proven that a diet devoid of fresh meats, milk, eggs, peas and beans will produce pellagra, and that a diet of fresh meat and vegetable proteins will cure the disease.

---

Dr. A. R. Ponton of the Post Sanitarium, Post, Texas, is spending two months visiting the clinics of the chief hospitals in Chicago, Philadelphia, Baltimore and New York. While away he will attend the Clinical Congress of American Surgeons, to be held in Boston, October 25th to 29th, inclusive. Dr. Ponton expects to return to Post about the 28th of November.

---

The Nueces County Medical Association has adopted a resolution calling for a general meeting to be called Friday night, December 3d, for the purpose of discussing county hospital plans.

---

The Southern Medical Association held its annual meeting at Dallas, November 8, 9, 10 and 11. This was one of the most successful meetings that the Association has ever held. Thirteen States and the District of Columbia were represented by prominent physicians and surgeons. The attendance was more than there has been present for years. Atlanta was selected as the place of meeting for next year. Dr. Robert Wilson, Jr., of Charleston, S. C., was elected President; Dr. Holman Taylor, of Fort Worth, Vice-President; Dr. Guy Hunter, of Baltimore, Second Vice-President, and Dr. Seale Harris, of Birmingham, Secretary-Treasurer.

